# Supplementary material for: Identification of photoperiod-induced specific miRNAs in the adrenal glands of Sunite sheep (Ovis aries)
Source: Front Vet Sci. 2022 Jul 22;9:888207. doi: 10.3389/fvets.2022.888207 (PMC9354845; doi:10.3389/fvets.2022.888207)
Supplement: Supplementary file 1 [file Data_Sheet_1.ZIP › Supplementary file/miRDeep2 file.zip!/miRDeep2 file/Novel_Structure/SPLP42A1/Novel_416.pdf]

Provisional ID	:	NC_019458.2_265
Score total	:	2.2
Score for star read(s)	:	-1.3
Score for read counts	:	0
Score for mfe	:	1.9
Score for randfold	:	1.6
Score for cons. seed	:	
Total read count	:	27
Mature read count	:	27
Loop read count	:	0
Star read count	:	0

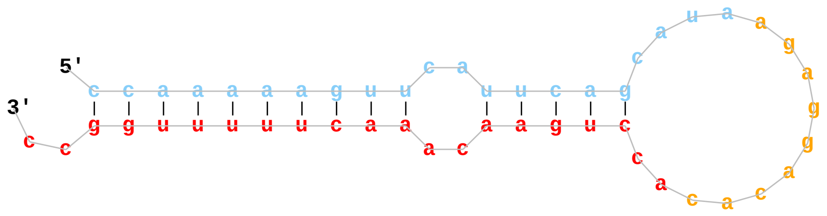

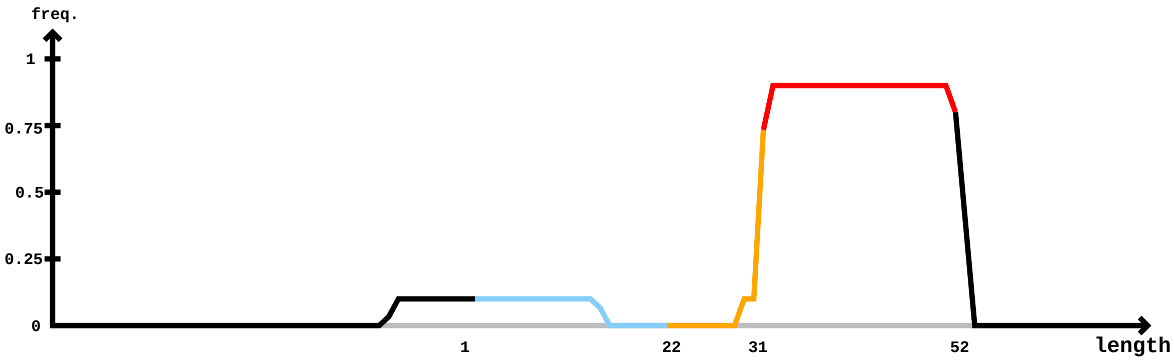

Star Mature

```
5'- aucacauuagugaagugcuaucccauagaaauauuggguuggccaaaaaguucauucagcauaagaggacacaccugaacaaacuuuuuggccaacccaguauaaagcaagc   -3'    exp
    .((((....))))..((((...........((((((((((((((((((((((..(((((...............)))))..))))))))))))))))))))))..))))...      reads      mm          sample
    .................................uuAgguuggccaaaaaguucau.........................................................      1          1           seq
    ..................................uAgguuggccaaaaaguucauu........................................................      1          1           seq
    ..................................uggguuggccaUaaaguucauu........................................................      1          1           seq
    ......................................................................acaccugaacaaacuuuuuggc....................      3          0           seq
    ........................................................................aUcugaacaaacuuuuuggcc...................      18         1           seq
    ........................................................................accugaacaaacuuuuuggcA...................      1          1           seq
    .........................................................................ccugaacaaacuuuuuggcU...................      1          1           seq
    .........................................................................Ucugaacaaacuuuuuggcca..................      4          1           seq
```
